# The Next Frontier of Remote Patient Monitoring: Hospital at Home

**DOI:** 10.2196/42335

**Published:** 2023-03-16

**Authors:** David Whitehead, Jared Conley

**Affiliations:** 1 Department of Emergency Medicine Massachusetts General Hospital Boston, MA United States; 2 Harvard Medical School Boston, MA United States; 3 Healthcare Transformation Lab Massachusetts General Hospital Boston, MA United States

**Keywords:** hospital at home, remote patient monitoring, RPM, digital health, remote monitoring, remote care, vital sign, telemetry, fall, cost, care delivery, service delivery

## Abstract

Remote patient monitoring (RPM) has shown promise in aiding safe and efficient remote care for chronic conditions; however, its use remains more limited within the hospital at home (HaH) model of care despite a significant opportunity to increase patient eligibility, improve safety, and decrease costs. HaH could achieve these goals by further adopting the 3 primary modalities of RPM (ie, vital sign, continuous single-lead electrocardiogram, and fall monitoring). With only 2 in-person vital sign checks required per day, HaH patient eligibility is currently often limited to lower-acuity cases. The use of vital sign RPM within HaH could better match the standard clinical practice of vital sign checks every 4-8 hours and enable safe care for appropriate moderate-acuity medical and surgical floor-level patients not traditionally enrolled in HaH. Robust, efficient collection of more frequent vital signs via RPM could expand patient eligibility for HaH and create a digital health safety net that enables high quality care. Similarly, our experience at Massachusetts General Hospital has demonstrated that appropriate use of continuous single-lead electrocardiogram RPM can also expand HaH enrollment, particularly for patients with acute decompensated heart failure. Through increasing enrollment of patients in HaH, RPM stands to enable more patients to reap the potential safety benefits of home hospitalization, including decreased rates of delirium and hospital-acquired infections, and better avoid aspects of posthospital syndrome. Furthermore, instituting fall detection RPM allows care teams to further HaH patient safety during their episode of acute care and develop enhanced mitigation strategies to avoid falls post home hospitalization. RPM also has the potential to assist HaH in achieving greater economies of scale and decreasing direct variable costs. By expanding HaH eligibility, RPM could enable HaH programs, which have traditionally operated under capacity, to care for a larger census and decrease allocated fixed costs per hospitalization. Additionally, RPM for HaH could further optimize hybrid in-home and remote nurse or physician evaluations, decreasing costs on a per-episode basis by up to an estimated 3.5%. Overall, RPM holds great promise to increase patient eligibility and patient safety while decreasing costs. However, it is in its infancy in achieving its potential to advance the HaH model of care; further research and experience that inform operational and technical as well as policy considerations are needed.

## Introduction

Over 250 hospitals and health systems are seeking to build or expand their hospital-level care at home, motivated largely by the Centers for Medicare and Medicaid Services’ (CMS) Acute Hospital Care at Home waiver, created during the COVID-19 pandemic to address hospital capacity issues, which allows for parity in Medicare reimbursement [1]. Recent extension of CMS reimbursement for hospital at home (HaH) through 2024 has further fueled interest and investment in HaH program development [2]. The HaH model in the United States was created in the 1990s and can provide care for patients with a growing range of acute medical conditions, including heart failure (HF) exacerbations, pneumonia, chronic obstructive pulmonary disease (COPD), and cellulitis, as well as postsurgical conditions.

Under the current CMS regulation, patients are enrolled in HaH after evaluation and management in the emergency department or from an inpatient medical or surgical hospital floor. Patients may also be admitted to HaH via other entry points depending on program structure and funding, including direct admission from home or outpatient clinics [3]. Studies demonstrate that the HaH model improves short- and long-term outcomes and saves costs compared to traditional hospitalization [4,5]. Experts estimate it could serve up to one third of US hospitalizations [4]. However, despite Medicare reimbursement catalyzing HaH implementation and investment in much-needed supporting infrastructure, HaH programs often continue to operate at a limited scale. Remote patient monitoring (RPM) has shown promise in aiding safe, effective, and more efficient chronic care management and represents a significant yet relatively unexplored opportunity for HaH to increase patient eligibility, improve patient safety, achieve operational efficiencies, and decrease costs [6,7].

## RPM for HaH

RPM refers to the collection of biometric data without hands-on clinical team involvement to inform clinical decision-making. RPM technologies for acute care can be classified by device modality used to collect biometric data and include ambient (ie, sensors using radar), wearable (ie, chest patch using one or a combination of photoplethysmography, electrocardiogram [ECG] electrodes, or accelerometers), or intermittent (ie, blood pressure cuff) devices [3]. Each device generally uses Bluetooth or similar technology to then transmit the biometric data to the clinical team. Primary variables of interest include vital signs, heart rhythm, and falls and can vary depending on the patient and their acute illness. Measurement frequency may also vary depending on the acuity of patient condition and clinical judgment.

## Opportunities for Remote Patient Monitoring for HaH

RPM implementation holds potential for HaH in several domains including (1) increasing patient eligibility, (2) improving patient safety, and (3) enhancing operational efficiencies and decreasing costs.

### Increasing Patient Eligibility

To date, many HaH programs have operated without using much RPM and have served predominantly the lowest-acuity patients in hospital wards, often only in urban settings. With RPM, HaH care could be provided for more patients with moderate-acuity medical and postsurgical conditions requiring frequent vital sign checks, continuous single-lead ECG, or fall monitoring, while expanding access in less population-dense communities [8].

**Figure 1:**
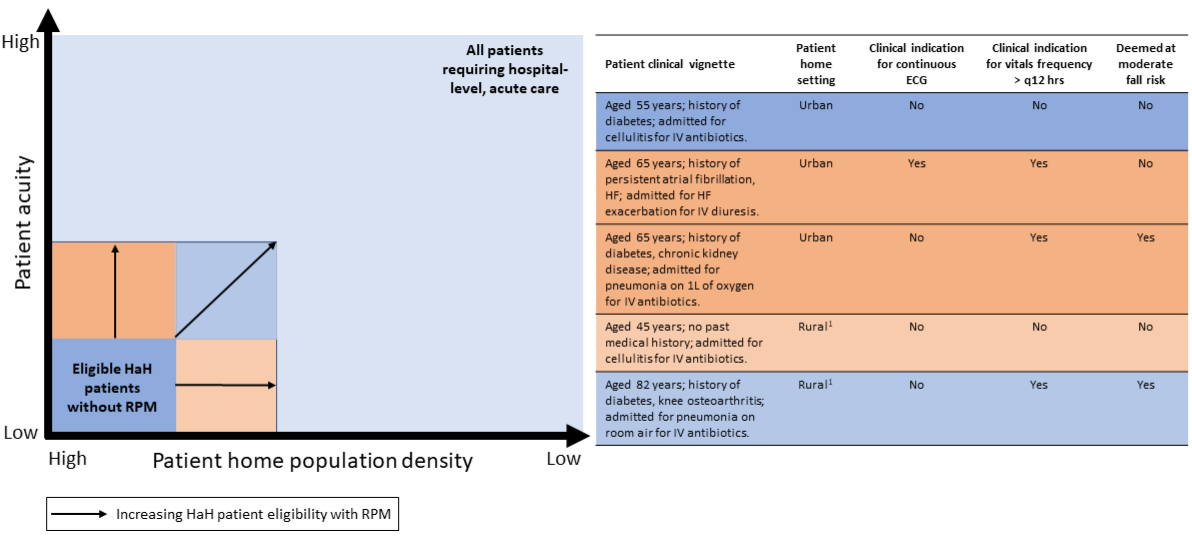
Potential for remote patient monitoring (RPM) to enhance hospital at home (HaH) patient eligibility. HF: heart failure; 1: potential candidate for tele-nurse remote evaluation with RPM.

Based on the HaH experience to date with often lower-acuity patients, CMS reimbursement policy requires 2 in-person evaluations per day by a nurse or paramedic during which patients’ vital signs are obtained. However, inpatient floor-level vital sign monitoring customarily is obtained every 4-8 hours, and data show as few as 5% of hospitalized patients have vital signs checked 2 or less times per day [9,10]. Limited data exist to inform optimal vital sign measurement frequency in hospitalized patients. However, studies point toward more frequent monitoring enabling earlier recognition of hospitalized patient decline and shorter hospital stays [11,12]. Additionally, a recent systematic review also identified a 39% decrease in risk of mortality and a trend toward less intensive care unit transfers and rapid response activations in hospital ward–level patients with multiparameter continuous vital sign monitoring compared to intermittent monitoring [13]. Yet more frequent, in-person vital sign collection can be a significant operational and costly burden for HaH. Therefore, streamlined, higher frequency or continuous vital sign evaluation via RPM could increase eligibility for HaH among patients who would otherwise be excluded due to their need for more frequent monitoring (Figure 1).

Remote monitoring of appropriate patients with continuous single-lead ECG is currently not a feature in many HaH programs but could significantly increase eligibility for patients with HF exacerbations—the second leading cause of nonmaternal, nonneonatal hospitalizations in the United States [14]. The American College of Cardiology recommends consideration of continuous ECG monitoring for patients with acute decompensated HF, paced rhythms, or chronic arrhythmias [15]. At Massachusetts General Hospital, we have used a continuous single-lead ECG device (VitalConnect) for the last 5-6 years in our HaH program and have good experience with it, expanding eligibility to hundreds of patients with acute decompensated HF, while ensuring their safety.

RPM use could also increase access to HaH services for suburban and rural populations. The CMS requirement for twice-daily check-ins can make HaH prohibitively costly to operate in less population-dense settings, where clinical teams may spend a disproportionate time enroute between patients. For appropriate low-acuity HaH patients, RPM could ease this constraint by replacing some in-home evaluations with a telenurse evaluation, making HaH more economically viable in rural settings.

### Improving Patient Safety

Although randomized controlled trials have shown HaH care to be as safe or safer when compared to inpatient hospital care, there is still significant room for improvement given continued safety issues still plaguing hospital care [4,16]. Through increasing enrollment of patients in HaH using RPM, more hospitalized patients may benefit from lower rates of delirium and limited exposure to hospital-acquired infections. In addition, they may better avoid aspects of posthospital syndrome, including becoming less deconditioned by bed rest or inactivity [17-19]. Hospitalizing patients in their homes, a familiar environment without entanglement hazards, such as wired monitoring devices, may also decrease the risk of falling, estimated to drive 84% of adverse hospital events [20]. Of course, the risk of falls cannot be completely removed during home hospitalization. Mitigation strategies, such as space modifications and assist devices, are appropriate and often already used in HaH. However, RPM for near-fall or fall detection can be used to further patient safety through constant monitoring of patients at appropriate risk (acknowledging that high-risk patients are perhaps best served in traditional inpatient settings) [21]. In our experience at Massachusetts General Hospital, applying RPM fall monitoring capabilities on appropriate patients, we have found that it has increased our detection of falls. It has also assisted us in promoting better long-term fall and injury avoidance for these patients by taking advantage of increased familiarity with their home setting to target fall mitigation interventions.

### Enhancing Operational Efficiencies and Decreasing Costs

HaH programs could use RPM to achieve greater economies of scale and decrease direct variable costs. HaH often requires significant administrative and infrastructure commitments, and HaH programs have traditionally operated under capacity. By expanding patient eligibility for HaH, RPM could decrease per-episode fixed cost allocation by enabling programs to run closer to capacity. To our knowledge, no literature exists evaluating the cost-effectiveness of RPM programs for HaH; however, studies evaluating RPM for chronic conditions, such as HF, COPD, and hypertension, have shown signs of cost-effectiveness [22]. Key drivers of cost-effectiveness identified for RPM for chronic illnesses include capital investment, clinical setting, and organizational processes involved in RPM implementation and delivery; therefore, programs should monitor expenditure closely and share best practices to mitigate unnecessary cost variation [22].

RPM could also potentially decrease costs of HaH care on a per-episode basis. We estimate using a cost analysis of Mount Sinai’s CMS HaH demonstration study that the average cost of a HaH episode could decrease by up to 3.5% by substituting RPM with telenurse evaluation for just 2 of the many in-person nursing evaluations during the multiday HaH care episode [5].

Additionally, HaH programs in conjunction with ambulatory clinicians could consider appropriate circumstances for patient continuation of RPM after HaH discharge to limit readmission risk. One health system that implemented RPM predominantly for patients with HF, COPD, and pneumonia after hospital discharge—a population similar to that cared for by HaH—reported a decrease in 30-day readmission from 14% to 6% and a decrease in total costs at 180 days post hospital discharge [23].

## Limitations and Risks of RPM for HaH

Effective RPM implementation for HaH will require a more robust understanding of the interplay between patients and acute pathology to determine the most clinically appropriate monitoring modality and frequency. In the interim, RPM may be under- and overused, and subsequently, it could generate unnecessary costs. Even when RPM is clinically indicated, it could lead to additional operational and financial burdens stemming from false alarms. Many early adopters are cognizant of this drawback and are developing methods to decrease them using artificial intelligence and other means [3]. Additionally, RPM devices may malfunction or fail. Automated remote efficacy and safety checks may mitigate this risk [24]. Lastly, RPM may not be the most effective method of monitoring some HaH patients. For example, patients deemed to be at a higher risk of falling might benefit most by a 24/7 in-home health aide to both prevent and monitor for a fall.

## A Proposed Road Map for Integrating RPM With HaH

Focus should be directed toward several key areas for RPM to achieve its potential to strengthen access to HaH services and further enhance the quality of HaH care (Table 1).

For RPM to deliver optimal value, robust research is needed to determine which RPM approach, if any, best serves a given HaH patient profile, as limited in-hospital research exists to inform monitoring modality and frequency. Second, to support RPM implementation, early adopters should be encouraged to openly share RPM best practices via forums such as the Hospital at Home Users Group and the World Hospital at Home Community. Additionally, clinicians and software engineers should continue to work together where RPM is implemented to ensure the data architecture and RPM alert pathways achieve safe and actionable physiologic monitoring, while minimizing false alerts. Lastly, policy makers should support RPM research and expansion efforts for HaH through enabling funding pathways, such as through a Center for Medicare and Medicaid Innovation demonstration project. Future Medicare reimbursement of HaH could also consider allowing for the substitution of some in-person visits with RPM-enabled telenurse evaluation for appropriate patients.

**Table 1:** Road map for remote patient monitoring (RPM) in hospital at home (HaH) care.

Domain	Priorities
Operational	Development of clinical guidelines to enable optimal matching of RPM (frequency and modality) to appropriate HaH patient profiles. Ensuring a robust RPM alert pathway exists that reaches appropriate clinical team member when significant vital sign deviation, fall, or dysrhythmia occurs. Advancing the implementation science of effective RPM use in HAH through research studies and increasing access to “working group” forums (eg, Hospital at Home Users Group and World Hospital at Home Community) for sharing and dissemination of best practices.
Technical	Further research evaluating as well as validating RPM device fidelity for monitoring of acutely ill, nonsedentary patients. Further development of reliable RPM alert software that ensures clinical team members are notified without fail. Standardization of data architecture to allow for robust EMR^a^ integration and rapid clinician decision-making. Further development of machine learning and artificial intelligence for improved detection of clinical deterioration and false alarm reduction.
Equity	Further development of robust LTE^b^ and Bluetooth-connected RPM devices along with form factor that enables compliance regardless of technology or health literacy as well as physical or cognitive impairments.
Policy	Center for Medicare and Medicaid Innovation demonstration projects evaluating the use of RPM for moderate-acuity HaH patients. Flexibility of CMS^c^ reimbursement to allow for 1 in-person registered nurse or paramedic visit and 1 RPM-enabled remote registered nurse evaluation per day for appropriate HaH patients.
Legal	Standardization and increased clarity of liabilities within contracts of third-party RPM device manufacturers, RPM software platforms, and health systems. Streamlined, clear informed consent process for patients deemed appropriate for HaH RPM.
Security	Development of HaH RPM security standards based on the National Cybersecurity Center of Excellence guidance on RPM and telehealth security.

^a^EMR: electronic medical record.

^b^LTE: long-term evolution.

^c^CMS: Centers for Medicare and Medicaid Services.

## Conclusions

As HaH continues to advance, broader use of RPM—guided by high-quality research and operational knowledge—has significant potential to enable more patients to benefit from the demonstrated value of healing in the comfort of one’s own home.

## Abbreviations

CMS: Centers for Medicare and Medicaid Services
COPD: chronic obstructive pulmonary disease
HaH: hospital at home
HF: heart failure
RPM: remote patient monitoring

## Data Availability

Data sharing is not applicable to this study, as no data sets were generated or analyzed during this study.

## References

[1] Acute hospital care at home resources. CMS QualityNet.

[2] Einhorn A, King O, Vernaglia L, Waltz J (2023). Acute hospital care at home: omnibus bill extends flexibility period to December 31,2024. National Law Rev.

[3] Conley J, Snyder GD, Whitehead D, Levine DM (2022). Technology-enabled hospital at home: innovation for acute care at home. NEJM Catalyst.

[4] Conley J, O'Brien CW, Leff BA, Bolen S, Zulman D (2016). Alternative strategies to inpatient hospitalization for acute medical conditions: a systematic review. JAMA Intern Med.

[5] (2017). Re: "HaH-Plus" (Hospital at Home Plus) provider-focused payment model. Icahn School of Medicine at Mount Sinai.

[6] Noah B, Keller MS, Mosadeghi S, Stein L, Johl S, Delshad S, Tashjian VC, Lew D, Kwan JT, Jusufagic A, Spiegel BMR (2018). Impact of remote patient monitoring on clinical outcomes: an updated meta-analysis of randomized controlled trials. NPJ Digit Med.

[7] Taylor ML, Thomas EE, Snoswell CL, Smith AC, Caffery LJ (2021). Does remote patient monitoring reduce acute care use? A systematic review. BMJ Open.

[8] Bryan AF, Levine DM, Tsai TC (2021). Home hospital for surgery. JAMA Surg.

[9] Orlov NM, Arora VM (2020). Things we do for no reason™: routine overnight vital sign checks. J Hosp Med.

[10] Ghosh E, Eshelman L, Yang L, Carlson E, Lord B (2018). Description of vital signs data measurement frequency in a medical/surgical unit at a community hospital in United States. Data Brief.

[11] Brown H, Terrence J, Vasquez P, Bates DW, Zimlichman E (2014). Continuous monitoring in an inpatient medical-surgical unit: a controlled clinical trial. Am J Med.

[12] Churpek MM, Adhikari R, Edelson DP (2016). The value of vital sign trends for detecting clinical deterioration on the wards. Resuscitation.

[13] Sun L, Joshi M, Khan SN, Ashrafian H, Darzi A (2020). Clinical impact of multi-parameter continuous non-invasive monitoring in hospital wards: a systematic review and meta-analysis. J R Soc Med.

[14] McDermott KW, Roemer M (2021). Most frequent principal diagnoses for inpatient stays in U.S. hospitals, 2018. Healthcare Cost and Utilization Project (HCUP) Statistical Briefs [Internet].

[15] Baibars M, Al-Khadra Y, Fanari Z, Moussa Pacha H, Soud M, Alraies MC (2018). Do all hospital inpatients need cardiac telemetry?. Cleve Clin J Med.

[16] Bates DW, Levine DM, Salmasian H, Syrowatka A, Shahian DM, Lipsitz S, Zebrowski JP, Myers LC, Logan MS, Roy CG, Iannaccone C, Frits ML, Volk LA, Dulgarian S, Amato MG, Edrees HH, Sato L, Folcarelli P, Einbinder JS, Reynolds ME, Mort E (2023). The safety of inpatient health care. N Engl J Med.

[17] Isaia G, Astengo MA, Tibaldi V, Zanocchi M, Bardelli B, Obialero R, Tizzani A, Bo M, Moiraghi C, Molaschi M, Ricauda NA (2009). Delirium in elderly home-treated patients: a prospective study with 6-month follow-up. Age (Dordr).

[18] Krumholz HM (2013). Post-hospital syndrome--an acquired, transient condition of generalized risk. N Engl J Med.

[19] Levine DM, Ouchi K, Blanchfield B, Saenz A, Burke K, Paz M, Diamond K, Pu CT, Schnipper JL (2020). Hospital-level care at home for acutely ill adults: a randomized controlled trial. Ann Intern Med.

[20] Aranda-Gallardo M, Morales-Asencio JM, Canca-Sanchez JC, Barrero-Sojo S, Perez-Jimenez C, Morales-Fernandez A, de LME, Moya-Suarez AB, Mora-Banderas AM (2013). Instruments for assessing the risk of falls in acute hospitalized patients: a systematic review and meta-analysis. BMC Health Serv Res.

[21] Kulurkar P, Dixit CK, Bharathi V, Monikavishnuvarthini A, Dhakne A, Preethi P (2023). AI based elderly fall prediction system using wearable sensors: a smart home-care technology with IOT. Measurement: Sensors.

[22] De Guzman KR, Snoswell CL, Taylor ML, Gray LC, Caffery LJ (2022). Economic evaluations of remote patient monitoring for chronic disease: a systematic review. Value Health.

[23] Siwicki B (2021). Deaconess health RPM program reduces cost of care by $7.4 million. Healthcare IT News.

[24] Simon DA, Cohen IG, Balatbat C, Offodile AC (2022). The hospital-at-home presents novel liabilities for physicians, hospitals, caregivers, and patients. Nat Med.

